# Efficacy of fluralaner administered either orally or topically for the treatment of naturally acquired *Sarcoptes scabiei* var. *canis* infestation in dogs

**DOI:** 10.1186/s13071-016-1670-7

**Published:** 2016-07-07

**Authors:** Janina Taenzler, Julian Liebenberg, Rainer K. A. Roepke, Régis Frénais, Anja R. Heckeroth

**Affiliations:** MSD Animal Health Innovation GmbH, Zur Propstei, Schwabenheim, 55270 Germany; ClinVet International, Uitsigweg, Bainsvlei, Bloemfontein, 9338 Free State South Africa; MSD Animal Health Innovation SAS, Beaucouzé Cedex, 49071 France

**Keywords:** Bravecto™, Bravecto™ Spot-on Solution, Chewable tablets, Fluralaner, Efficacy, Dog, Oral, *Sarcoptes scabiei* var. *canis*, Topical, Mange, Mites

## Abstract

**Background:**

The efficacy of fluralaner, formulated as a chewable tablet (Bravecto™) or topical solution (Bravecto™ Spot-on Solution), was evaluated against naturally acquired *Sarcoptes scabiei* var. *canis* infestation in dogs.

**Methods:**

The study was performed in privately-owned dogs naturally infested with *S. scabiei* var. *canis.* All dogs living in the same household as the infested dog were enrolled into one of 3 groups (2 fluralaner treated and 1 negative control). All dogs within one household were administered the same treatment, with one dog per household included in further observations and assessments. In total, 29 dogs confirmed positive for sarcoptic mange were included. On Day 0, all dogs in group 1 (*n* = 9) were treated once orally with fluralaner at a minimum dose of 25 mg/kg body weight; all dogs in group 2 (*n* = 11) were treated once topically with fluralaner at a dose of 25 mg/kg body weight; and dogs in group 3 (*n* = 9) were treated once topically with saline solution. *Sarcoptes scabiei* var. *canis* mites on each dog were counted before treatment and at 4 weeks after treatment in deep skin scrapings (~4 cm^2^) from 5 different body areas. Clinical signs of infestation (i.e. erythematous papules; casts, scales and crusts; body areas with hair loss) and pruritus were recorded at the same time points.

**Results:**

Single oral or topical treatment with fluralaner resulted in a 100 % reduction in mite counts post-treatment (group 1: *P* = 0.0009 and group 2: *P* = 0.0011). Resolution of clinical signs at four weeks post-treatment was variable, with improvement observed for erythematous papules, casts and crusts, and pruritus. All fluralaner treated dogs showed an improvement in overall hair re-growth compared with pre-treatment observations.

**Conclusion:**

Fluralaner administered either orally or topically to naturally infested dogs eliminates *Sarcoptes scabiei* var. *canis* mites and improves clinical signs over a 4-week observation period.

## Background

Ectoparasites are a common cause of dermatological diseases in dogs. Among these, an infestation with *Sarcoptes scabiei* is one of the most frequent mite infestations worldwide [1]. *S. scabiei* is a burrowing mite, infesting not only dogs, but also cats [2], pigs [3], raccoon dogs [4], rabbits [5], sheep [6], and humans [7]. The mites preferentially inhabit less hairy regions of the host’s body, and the severity of clinical signs and mite infestation on skin surface areas may differ from one host species to another [8]. The mite causing sarcoptic mange in the dog is *S. scabiei* var. *canis.* Sarcoptic mange is highly contagious and highly pruritic, making it one of the most uncomfortable skin diseases of the dog. Infestations with *S. scabiei* var. *canis* are non-seasonal, without any age, breed or sex predilection and occur by direct contact with an infested dog or by contact with infested dog’s bedding [1]. The clinical signs include intense constant pruritus, erythematous rash, papules and yellowish crusts that form on the skin surface, together with alopecia [9]. The most affected skin areas are the periocular skin, ear pinna, elbows, and hocks, with spread to other areas over time [1]. In North American veterinary teaching hospitals, sarcoptic mange is one of the most commonly diagnosed skin diseases [10]. Definitive diagnosis of *S. scabiei* relies on the microscopic demonstration of mites and their eggs on skin scrapings [11] although observation of mites may not be easy.

Currently available licensed therapeutic options for sarcoptic mange may include the active compounds selamectin [12], imidacloprid/moxidectin [13] and in some countries amitraz [14], which are administered topically. A recent therapeutic option in some areas is oral treatment with the isoxazoline sarolaner. This compound may need to be administered more than once to eliminate the mites [15]; therefore, requiring owner administration compliance over an extended period.

Fluralaner is an isoxazoline ectoparasiticide that provides an extended period of persistent efficacy against ticks and fleas for dogs [16]. A single administration of fluralaner is also highly effective against generalized demodicosis in dogs [17]. In this study, the efficacy of a single fluralaner treatment (Bravecto™), formulated as either a chewable tablet or as a spot-on solution was evaluated for the treatment of naturally acquired *Sarcoptes scabiei* var. *canis* infestation in dogs.

## Methods

### Study set-up

The study set-up was designed as a parallel group, blinded, randomized, and controlled efficacy design conducted in the republic of South Africa. Procedures were in accordance with Good Clinical Practice (VICH guideline GL9, Good Clinical Practice, EMA, 2000). Masking of the study personnel was assured through the separation of study functions. All personnel conducting observations or animal care or performing mite examinations and counts after treatment were masked to treatment allocation.

### Inclusion criteria

Dogs were included, if they were naturally infested with *S. scabiei* var. *canis* as confirmed by skin scrapings, were healthy on physical examination except for visible clinical signs associated with a sarcoptic mange infestation, e.g. constant pruritus, alopecia, erythematous rash and yellowish crusts on the affected skin areas, did not harbour any *Demodex* spp. mites, and were not treated with any product with an acaricidal/insecticidal effect for at least 8 weeks prior to treatment.

When a dog in a household met the inclusion criteria, then the complete household was enrolled in the study and randomly allocated to one of 3 study groups (2 fluralaner treated and 1 negative control). All dogs in each enrolled household were administered the same treatment, but only the dog that met the inclusion criteria was included in further observations, assessments and efficacy calculations. Consent of the dog owner was obtained before study inclusion.

### Animal details

In total, 29 dogs (19 male and 10 female) were included in the study. All dogs were mixed breed (mainly mongrels), older than 6 months, weighing between 5.6 and 25.3 kg at the day of treatment, and female dogs were not clinically pregnant or lactating. For the duration of the study, each dog stayed with its owner under its usual housing conditions. Feeding and potable water supply was continued according to the owner’s prior preferences and contact with other animals was not restricted.

### Treatment

On Day 0 (i.e. day of treatment), dogs in group 1 (*n* = 9) were treated once orally with fluralaner chewable tablets at the minimum dose of 25 mg fluralaner/kg body weight. Individual oral doses were determined on the basis of the dog’s individual body weight and the nominal content of fluralaner in the tablets. Dogs received whole tablets using either 112.5 mg, 250 mg or 500 mg fluralaner tablets, or a combination of tablets to achieve a dose close to the calculated target dose. The tablet(s) were administered 20 (±10) minutes after food had been offered by placement in the back of the oral cavity over the tongue to initiate swallowing. No vomit or regurgitation was observed in any treated dog. Dogs in group 2 (*n* = 11) were treated once topically with fluralaner spot-on solution at a dose of 25 mg fluralaner/kg body weight. Dogs in group 3 (*n* = 9) were treated once topically with saline solution at a volume of 0.09 ml/kg body weight. A negative control group was included to prevent bias in personnel performing mite assessments. Dogs in groups 2 and 3 were treated only at administration sites that were free of any observed skin lesions. Topical administration was performed with the dog in a standing position, at one or more spots along the dog’s dorsal line, from the shoulder blades to the base of the tail, depending on the total administration volume. Hair was parted and the tip of the disposable syringe was placed vertically on the skin and the solution/saline administered directly to the skin by pressing the plunger of the syringe to empty its contents. No evidence of mis-dosing, such as spillage or run-off/drip-off, was reported in any treated animal.

### Mite assessments

Deep skin scrapings (~4 cm^2^), involving squeezing and then scraping the skin until capillary oozing was seen, were made from 5 different body areas showing clinical signs of sarcoptic mange before treatment. Dogs with mite positive scrapings were eligible for inclusion, and scrapings were repeated 4 weeks after treatment, to measure treatment efficacy. Each scraping was transferred to a separate labelled microscope slide containing mineral oil and was examined under a stereomicroscope for presence of live *S. scabiei* var. *canis* mites. The numbers of live mites were counted in each scraping.

### Skin and pruritus assessments

The clinical signs and the extent of sarcoptic lesions on each dog were assessed pre-treatment and at 4 weeks post-treatment. The following parameters, sketched on a silhouette (left and right hand side of the dog), were assessed for each dog: body areas exhibiting erythematous papules; body areas covered by casts, scales and crusts; body areas with hair loss (alopecia). In addition, the presence or absence of pruritus was assessed by observing the dog for 5 min.

### Data analysis

The statistical analysis to evaluate the efficacy was performed using the software package SAS® (SAS Institute Inc., Cary, NC, USA, release 9.3), with the individual dog as statistical unit. The primary assessment variable in the study was the total number of mites counted in skin scraping following treatment. The percentage of efficacy against *S. scabiei* var. *canis* mites was calculated using geometric means with Abbott’s formula:

Efficacy (%) = 100 × (M_C_ – M_T_), where M_C_ is the geometric mean number of total mite counts in the control group (group 3), and M_T_ the geometric mean number of total mite counts in the treatment group (group 1 or 2). Log-transformed [ln(x + 1)] counts of *S. scabiei* mites were used to confirm the efficacy calculation. Significant differences were assessed between the log-counts of *S. scabiei* var. *canis* mites in the treated groups compared to the log-counts of the control group using a linear mixed model including study group as a fixed effect and block as a random effect. The two-sided level of significance was set *P* ≤ 0.05 (One-way ANOVA with a treatment effect).

The success rate, i.e. number of dogs without mites 4 weeks post-treatment, was calculated as follows: Success Rate (n) = number of dogs with an absence of live mite counts in the group/total number of dogs in the group.

The secondary assessment variable in the study was the resolution of clinical signs, which were assessed descriptively by comparing pre- and post-treatment observations for each parameter. Regarding hair loss, a semi-quantitative assessment of hair re-growth was performed (body areas with hair re-growth 0–50 %; body areas with hair re-growth 50–90 % or body areas with hair re-growth > 90 %).

## Results

No adverse event related to fluralaner treatment was observed in any dog during the 4 week post-treatment observation period. However, 3 dogs treated topically with fluralaner, did not complete the study. One dog was killed by community members and, 2 others died. The cause of death was not ascertained but it is likely that a secondary infection associated with sarcoptic mange was responsible [18].

In six out of 9 control dogs mite positive skin scrapings were obtained 4 weeks post-treatment (mean mite count 7.9), whereas in 3 dogs no mites were present. In any orally or topically fluralaner treated dog, no mites were found in any skin scraping obtained 4 weeks post-treatment resulting in 100 % efficacy for both groups (group 1: *P* = 0.0009 and group 2: *P* = 0.0011; Table 1). All treated dogs were free of mites, thus a 100 % success rate was achieved.

**Table 1:** Mean mite counts and efficacy (%) after single oral or topical administration of fluralaner to dogs naturally infested with *Sarcoptes scabiei* var. *canis*

Assessment time point	Treatment	Saline solution	Fluralaner chewable tablets	Fluralaner spot-on solution
		*n* = 9	*n* = 9	*n* = 8
Pre-treatment	Mean^a^ mite counts (n)	10.6	12.0	13.1
	Count range (n)	2–34	2–163	2–67
4 weeks post-treatment	Mean^a^ mite counts (n)	7.9^b^	0	0
	Count range (n)	1–55^b^	0	0
	Efficacy (%)	na	100	100
	*P*-value^c^	na	0.0009	0.0011

*n* number of dogs per group, *na* Not applicable

^a^Geometric mean

^b^Dogs with zero mite counts are excluded

^c^One-way ANOVA with a treatment effect

Observed changes in clinical signs in all groups were variable (Table 2). All dogs treated topically with fluralaner (*n* = 8) showed no casts, crusts and erythematous papules at 4 weeks post-treatment. The number of dogs presented with scales increased from 5 pre-treatment to 7 post-treatment, whereas pruritus was only present in 1 dog post-treatment compared to 4 dogs pre-treatment.

**Table 2:** Dermatological signs in dogs with sarcoptic mange before fluralaner treatment and four weeks thereafter

Treatment	Fluralaner chewable tablets (number of dogs/number of dogs per group)	
Clinical sign	Pre-treatment	4 weeks post-treatment
Crusts	7/9 (78 %)	3/9 (33 %)
Casts	0/9 (0 %)	0/9 (0 %)
Scales	4/9 (44 %)	7/9 (78 %)
Erythematous papules	3/9 (33 %)	2/9 (22 %)
Pruritus	5/9 (56 %)	2/9 (22 %)
Treatment	Fluralaner spot-on solution (number of dogs/number of dogs per group)	
Clinical sign	Pre-treatment	4 weeks post-treatment
Crusts	6/8 (75 %)	0/8 (0 %)
Casts	1/8 (13 %)	0/8 (0 %)
Scales	5/8 (63 %)	7/8 (88 %)
Erythematous papules	1/8 (13 %)	0/8 (0 %)
Pruritus	4/8 (50 %)	1/8 (13 %)

After oral fluralaner treatment (*n* = 9), clinical signs resolved in 4 (crusts), 1 (erythematous papules) and 3 (pruritus) dogs. The number of dogs presented with scales increased from 4 pre-treatment to 7 post-treatment.

Hair re-growth at 4 weeks post-treatment was apparent in all orally or topically fluralaner treated dogs (Table 3).

**Table 3:** Hair re-growth on dogs with sarcoptic mange after oral or topical treatment with fluralaner

Assessment time point	Estimated percent hair re-growth^a^					
	Fluralaner chewable tablets (number of dogs/number of dogs per group)			Fluralaner spot-on solution (number of dogs/number of dogs per group)		
	0–50 %	50–90 %	>90 %	0–50 %	50–90 %	>90 %
4 weeks post-treatment	6/9	1/9	2/9	4/8	4/8	0/8

^a^Percentage hair re-growth defined as estimated percentage of hair cover growth at 4 weeks post-treatment compared to baseline total hairless area assessed prior to fluralaner administration

## Discussion

Fluralaner administered orally or topically, is highly effective against naturally acquired *Sarcoptes scabiei* var. *canis* infestation in dogs. There was no apparent difference in efficacy between the administration routes. Fluralaner’s efficacy against *Sarcoptes* mites is consistent with reported efficacy of orally administered fluralaner against *Demodex* mite infestation in dogs [17]. Elimination of mites led to apparent hair re-growth over the 4 weeks post-treatment. However, resolution of clinical signs was variable in both fluralaner treated groups, and it is possible that dead mites remaining in the skin continued to cause local irritation over the 4 week post-treatment period.

In 3 out of 9 control dogs, no mites in skin scrapings at 4 weeks post-treatment were observed, indicating an apparent self-clearing of the mite infestation. Difficulties in finding mites in skin scrapings from dogs with clinical signs of sarcoptic mange has been observed, especially when the patient is intensively pruritic and has had the disease for a long time or has received multiple baths or dips [8]. The apparent self-clearing in control animals has also been observed in other laboratory studies with untreated controls [19] or placebo-treated animals [15, 20]. Placebo-treated dogs, that also received immunosuppressive treatment throughout the study period, maintained their mite infestation [15]. The difficulty of finding mites in skin scrapings can affect the efficacy calculations; however, use of immunosuppressive therapy for dogs in a field study is not acceptable.

Complete elimination of mites, due to fluralaner treatment, also led to a reduction in the number of dogs with pruritus. A sarcoptic mange infestation is intensely pruritic, creating high discomfort for the infested dog due to the urge of continuous scratching. Even dead mites continue to cause intense pruritus and it takes some time after successful treatment for pruritus to resolve and for associated skin lesions to disappear. The increased number of dogs shedding scales is presumably related to the skin’s healing process.

In this study the 4 weeks between treatment and assessment of skin lesions was likely too short to allow complete resolution of dermatological lesions. Fluralaner treatment provides systemic ectoparasiticide efficacy for up to 12 weeks against ticks and fleas on dogs [16, 21], and therefore fluralaner administration should provide sustained control of *Sarcoptes* mite infestations in susceptible dogs following treatment.

## Conclusion

Fluralaner administered either orally or topically to naturally infested dogs eliminates *Sarcoptes scabiei* var. *canis* mites and improves clinical signs over a 4 week observation period.
